# Low risk of local recurrence after a successful en bloc endoscopic submucosal dissection for noninvasive colorectal lesions with positive horizontal resection margins (R-ESD study)

**DOI:** 10.1055/a-1960-3552

**Published:** 2023-01-12

**Authors:** Krijn J. C. Haasnoot, Francisco Baldaque-Silva, Arjun Koch, Mariana Figueiredo Ferreira, João Santos-Antunes, Emanuel Dias, Masami Omae, Laurelle van Tilburg, Hao Dang, Arnaud Lemmers, Jurjen J. Boonstra, Leon M. G. Moons

**Affiliations:** 1 Department of Gastroenterology and Hepatology, University Medical Center Utrecht, Utrecht, The Netherlands; 2 Endoscopy Unit, Center for Upper Digestive Diseases, Karolinska University Hospital and Karolinska Institute, Stockholm, Sweden; 3 Pedro Hispano Hospital, Advanced Endoscopy Center Carlos Moreira da Silva, Porto, Portugal; 4 Department of Gastroenterology and Hepatology, Erasmus MC Cancer Institute, University Medical Center, Rotterdam, The Netherlands; 5 Department of Gastroenterology, Hepatopancreatology and Digestive Oncology, Erasme Hospital, Université Libre de Bruxelles, Brussels, Belgium; 6 Department of Gastroenterology and Hepatology, University Hospital Center of São João, Porto, Portugal; 7 IPATIMUP/i3S, University of Porto, Porto, Portugal; 8 Department of Gastroenterology and Hepatology, Leiden University Medical Center, Leiden, The Netherlands

## Abstract

**Background** During endoscopic submucosal dissection (ESD), the normal mucosa is cut under constant optical control. We studied whether a positive horizontal resection margin after a complete en bloc ESD predicts local recurrence.

**Methods** In this European multicenter cohort study, patients with a complete en bloc colorectal ESD were selected from prospective registries. Cases were defined by a horizontal resection margin that was positive or indeterminate for dysplasia (HM1), whereas controls had a free resection margin (HM0). Low risk lesions with submucosal invasion (T1) and margins free of carcinoma were analyzed separately. The main outcome was local recurrence.

**Results** From 928 consecutive ESDs (2011–2020), 354 patients (40 % female; mean age 67 years, median follow-up 23.6 months), with 308 noninvasive lesions and 46 T1 lesions, were included. The recurrence rate for noninvasive lesions was 1/212 (0.5 %; 95 %CI 0.02 %–2.6 %) for HM0 vs. 2/96 (2.1 %; 95 %CI 0.57 %–7.3 %) for HM1. The recurrence rate for T1 lesions was 1/38 (2.6 %; 95 %CI 0.14 %–13.5 %) for HM0 vs. 2/8 (25 %; 95 %CI 7.2 %–59.1 %) for HM1.

**Conclusion** A positive horizontal resection margin after an en bloc ESD for noninvasive lesions is associated with a marginal nonsignificant increase in the local recurrence rate, equal to an ESD with clear horizontal margins. This could not be confirmed for T1 lesions.

## Graphical abstract

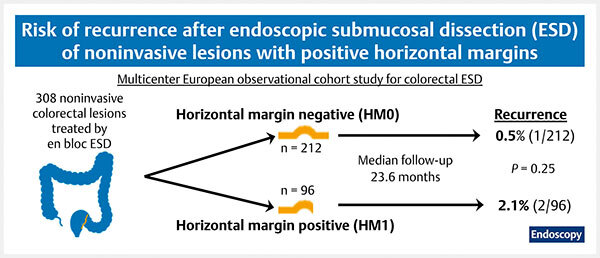

## Introduction

A positive or indeterminate horizontal resection margin (HM1) after polypectomy of a colorectal polyp warrants intensive endoscopic follow-up as it is associated with an increased risk of local recurrence 1 . Because neoplasia and normal mucosa are distinguishable, colorectal endoscopic submucosal dissection (ESD) has the advantage of continuous optical control during the mucosal cut. Furthermore, HM1 after ESD may be caused by cauterization, a suboptimal embedding technique, or tangential cutting in the pathology department 2 3 .

A pathologist’s judgement of the horizontal margin may perhaps not be superior to that of an endoscopist by virtue of the fact that, with ESD, the endoscopist visualizes the cut through normal mucosa. This makes it unclear whether HM1 imposes a risk of local recurrence after ESD in patients with an en bloc resection. Based on low quality evidence, the European Society of Gastrointestinal Endoscopy guideline for ESD advises colonoscopy at 3–6 months after HM1 4 . More recently, an Asian study with a recurrence rate of 1.5 % after ESD found an increased risk after histologic incomplete resection and also advised first follow-up should be after 3–6 months 5 . On the other hand, HM1 after complete ESD was not predictive of recurrence during follow-up in a second Asian study; however, the number of cases was too low to draw strong conclusions 6 .

We hypothesized that the recurrence rate after complete ESD for colorectal lesions with positive horizontal resection margins (HM1) would not be increased compared with that for lesions with free resection margins (HM0).

## Methods

### Study design and patient selection

This study was a multicenter multinational observational cohort study. Patients were identified from individual prospective ESD databases in six centers that were specialized in the endoscopic resection of large colorectal neoplasia: Karolinska Institute (Stockholm, Sweden), Erasme Hospital (Brussels, Belgium), São João University Hospital Center (Porto, Portugal), and three Dutch centers: Erasmus MC (Rotterdam), Leiden University Medical Center (LUMC), and University Medical Center Utrecht (UMCU).

The inclusion criteria were: a complete ESD for a colorectal lesion, defined as an en bloc and macroscopic radical resection, as judged by the endoscopist. ESD was indicated either as treatment for a recurrent adenoma after prior polypectomy, or as the first treatment for lesions with features suspicious for superficial invasive carcinoma on optical diagnosis or for rectal polyps larger than 20 mm irrespective of any features of superficial invasion. Patients needed to have had one or more follow-up endoscopies for scar inspection. Cases were excluded if there was a positive vertical resection margin mentioned, a nonadenoma origin, or if the scar site was resected. Lesions with submucosal invasion were included if the horizontal and vertical resection margins were free of carcinoma and when high risk features (high grade tumor budding, lymphovascular invasion, poor differentiation grade, or positive resection margin for carcinoma) were absent (“low risk T1 CRC”).

Consecutive patients meeting the inclusion and exclusion criteria were selected from the prospective databases from 2011 to 2020. The study was approved by the local medical ethics committees of all centers: UMCU (19–228/C), LUMC (G18.097/SH/sh), Erasme Hospital (P2020/186), Karolinska (2020–05737), and São João (255/2020).

### Patient, lesion, and ESD characteristics

Patient characteristics were extracted from the electronic medical records. Information on lesion characteristics (size, location, Paris classification, circumferential involvement) and procedural parameters (access to the polyp, intraprocedural bleeding, duration) were extracted from standardized endoscopy reports. The difficulty of the procedure (easy/moderate/difficult) was subjectively assessed by the performing endoscopist. Histological parameters were extracted from pathology reports. The proximal colon was defined as any lesion proximal to (and including) the splenic flexure. The distal colon was defined as the sigmoid and descending colon.

### Histology

As part of the routine practice in all participating hospitals, all cases were reviewed by expert gastrointestinal pathologists and reported according to the WHO classification guidelines 7 . For HM1 resections, cases were discussed and reclassified if needed. Patients with a horizontal resection margin containing dysplasia or indeterminate margins were identified as cases (HM1), and patients with horizontal resection margins that were free of dysplasia as controls (HM0).

### Follow-up

Data on the outcomes of scar inspection were retrospectively extracted from electronic medical records, and by contacting the referral hospital. In the latter case, endoscopy images, as well as the histology of scar biopsies, were retrieved and reviewed for the presence of recurrence. The follow-up regimen was unstandardized.

The duration of follow-up was defined as the last follow-up endoscopy with scar inspection or the detection of histologically confirmed recurrence.

### Outcomes

The main outcome was histologically confirmed recurrence detected within 5 mm of the scar during follow-up, thereby conforming with the Higaki recurrence criteria 8 . If no recurrent lesion was found on endoscopic evaluation, it was left to the discretion of the endoscopist to perform biopsies of the normal-looking scar tissue. Local recurrence had to be confirmed by histologic evaluation (dysplastic tissue) of biopsies or resection of the lesion.

The recurrence rate is shown for HM0 and HM1, and is assessed separately for noninvasive and T1 lesions.

### Statistical analysis

The recurrence rate after ESD was 0–2 % in Asian studies 9 . To show that the upper 95 %CI limit would not exceed 5 % with an expected recurrence proportion of 2 %, a sample of 96 patients was needed in both arms. We aimed to have follow-up with endoscopy of at least 6 months for all included patients as a meta-analysis for recurrence after endoscopic mucosal resection (EMR) reported that 96 % of recurrences were detected at 6 months and 98 % at 12 months 1 . Categorical variables were compared by chi-squared test and continuous variables by one-way ANOVA or Kruskal–Wallis test as appropriate. The relative risks with corresponding 95 %CIs were calculated using Wald intervals.

The recurrence rates for HM0 and HM1 resected cases were compared with an odds ratio (OR) over time by applying a generalized estimating equation to take repeated observations within patients into account. Survival analyses were estimated with the Kaplan–Meier method. *P* < 0.05 was considered statistically significant. The Wilson score was the method used to calculate 95 %CI for proportions.

IBM SPSS Statistics version 26 and R version 4.0.3 were used for the statistical analyses.

## Results

### Study population

**Fig. 1:**
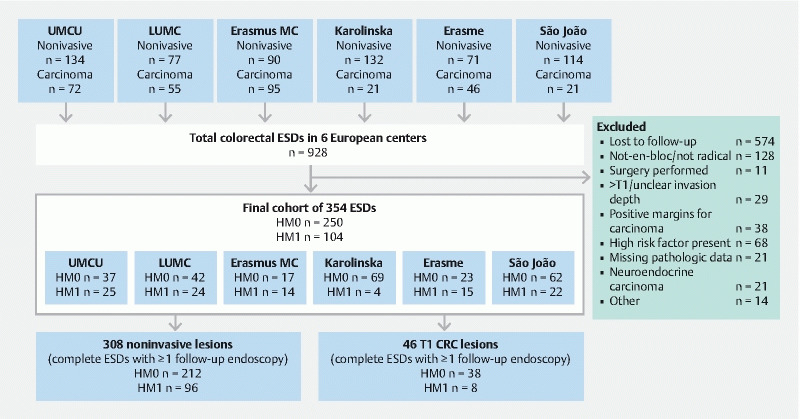
Flowchart of all endoscopic submucosal dissection (ESD) procedures selected. HM1, horizontal resection margin positive or indeterminate for dysplasia; HM0, a free horizontal resection margin; CRC, colorectal carcinoma; UMCU, University Medical Center Utrecht; LUMC, Leiden University Medical Center.

From a total of 928 consecutive cases, 354 cases fulfilled our inclusion criteria and were selected from the prospective registries ( Fig. 1 ). Patient and polyp characteristics are provided in Table 1 .

**Table 1:** Descriptive characteristics of the study population and risk factors for an HM1 resection margin.

	All ESD procedures (n = 354)	HM0 resections (n = 250)	HM1 resections (n = 104)	*P* value	Relative risk for HM1	95 %CI
Sex, n (%)						
Female	143 (40.4)	94 (37.6)	49 (47.1)	0.12	Reference	
Male	211 (59.6)	156 (62.4)	55 (52.9)		0.76	0.55–1.05
Age, median (IQR), years	67 (60–74)	67 (59–73)	69 (61–75)	0.13		
Clinical indication for ESD, n (%)						
Suspected superficial invasive carcinoma 1	345 (97.5)	246 (98.4)	99 (95.2)	0.17	Reference	
Recurrent lesion	9 (2.5)	4 (1.6)	5 (4.8)		1.94	1.05–3.55
Location of the lesion, n (%)						
Proximal colon	42 (11.9)	35 (14.0)	7 (6.7)	0.16	Reference	
Distal colon	52 (14.7)	36 (14.4)	16 (15.4)		1.85	0.84–4.07
Rectum	260 (73.4)	179 (71.6)	81 (77.9)		1.87	0.93–3.76
Histology, n (%)						
LGD	168 (47.5)	120 (48.0)	48 (46.2)	0.13	Reference	
LGD with focal HGD	68 (19.2)	46 (18.4)	22 (21.2)		1.13	0.75–1.72
HGD	69 (19.5)	43 (17.2)	26 (25.0)		1.32	0.90–1.94
Serrated lesion	3 (0.8)	3 (1.2)	0 (0.0)		–	–
T1 CRC	46 (13.0)	38 (15.2)	8 (7.7)		0.61	0.31–1.19
Polyp size on endoscopy report, median (IQR), mm	40 (30–50)	35 (30–50)	45 (30–65)	< 0.001		
Polyp size on pathology report, median (IQR), mm	40 (27–55)	35 (26–49)	50 (40–65)	< 0.001		
Paris classification, n (%)						
0-IIa	84 (23.7)	60 (24.0)	24 (23.1)	0.81	Reference	
0-IIa + Is	114 (32.2)	77 (30.8)	37 (35.6)		1.14	0.74–1.75
0-IIa + c	21 (5.9)	16 (6.4)	5 (4.8)		0.83	0.36–1.92
0-IIb	5 (1.4)	4 (1.6)	1 (1.0)		0.70	0.12–4.17
0-IIc	8 (2.3)	7 (2.8)	1 (1.0)		0.44	0.07–2.82
Is	115 (32.5)	80 (32.0)	35 (33.7)		1.07	0.69–1.65
Isp	7 (2.0)	6 (2.4)	1 (1.0)		0.50	0.08–3.17
Circumference of the lumen, n (%)						
< 50 %	133 (59.9)	97 (66.4)	36 (47.4)	0.009	Reference	
≥ 50 %	89 (40.1)	49 (33.6)	40 (52.6)		1.66	1.16–2.38
Missing	132	104	28			
Access to the polyp, n (%)						
Easy	116 (57.4)	98 (62.8)	18 (39.1)	< 0.001	Reference	
Moderate	61 (30.2)	49 (31.4)	12 (26.1)		1.27	0.65–2.46
Difficult	25 (12.4)	9 (5.8)	16 (34.8)		4.12	2.46–6.91
Not reported	152	94	58			
Intraprocedural bleeding needing intervention, n (%) 2						
No/not described	317 (89.5)	230 (92.0)	87 (83.7)	0.03	Reference	
Yes	37 (10.5)	20 (8.0)	17 (16.3)		1.67	1.13–2.48
Duration of the ESD, mean (SD), minutes	127 (81)	113 (68)	162 (96)	< 0.001		

ESD, endoscopic submucosal dissection; HM1, horizontal resection margin positive or indeterminate for dysplasia; HM0, free horizontal resection margin; IQR, interquartile range; LGD, low grade dysplasia; HGD, high grade dysplasia; CRC, colorectal carcinoma.

1 Lesions with features suspicious for superficial invasive carcinoma based on either optical diagnosis or rectal polyps larger than 20 mm irrespective of features of superficial invasion.

2 Additional intraprocedural interventions, such as the use of coagulation forceps or hemoclips.

### Risk factors for HM1

An HM1 resection was significantly associated with larger size lesions, ≥ 50 % circumferential involvement, difficult access to the polyp, intraprocedural bleeding, and longer procedure duration ( Table 1 ). There was no learning curve for HM1 during the study cohort.

### Risk of recurrence after an HM1 resection margin

The median follow-up time for noninvasive lesions was 23.9 (IQR 9.34–37.4) months and was equal for HM0 vs. HM1 ( *P* = 0.46) ( Table 2 ). However, follow-up time was significantly longer for T1 lesions with HM0 than with HM1 (27.4 vs. 9.2 months; *P* = 0.001) ( **Table 1 s** , see online-only Supplementary Material).

**Table 2:** Recurrence during follow-up of HM0 vs. HM1 for noninvasive lesions.

**Follow-up time and detected recurrence**						
Noninvasive lesions	Follow-up time, median (IQR), months	*P* value	Recurrence detected during follow-up, n/N (%)	95 %CI for proportion 1	Odds ratio (95 %CI) 2	*P* value
Total	23.9 (9.34–37.4)					
HM0	25.7 (11.2–37.7)	0.46	1 /212 (0.5 %)	0.02 %–2.6 %	0.24 (0.02–2.74)	0.25
HM1	21.8 (7.97–36.8)		2/96 (2.1 %)	0.57 %–7.3 %	Reference	
**Survival analysis**						
Noninvasive lesions	6 months		12 months		36 months	
HM0						
Recurrence/patients at risk	0/187		0/154		1/67	
Recurrence free survival (95 %CI)	100 % (100 %–100 %)		100 % (100 %–100 %)		98.9 % (97 %–100 %)	
HM1						
Recurrence/patients at risk	0/81		0/63		2/30	
Recurrence free survival (95 %CI)	100 % (100 %–100 %)		100 % (100 %–100 %)		95.9 % (90 %–100 %)	

HM1, horizontal resection margin positive or indeterminate for dysplasia; HM0, a free horizontal resection margin; IQR, interquartile range.

1 95 %CI for proportion calculated with the Wilson score method.

2 Odds ratio calculated from generalized estimating equation.

Three recurrences were observed in the noninvasive group, of which one occurred in HM0 and two in HM1 (0.5 % [95 %CI 0.02 %–2.6 %] vs. 2.1 % [95 %CI 0.57 %–7.3 %]). The odds ratio over time for HM0 vs. HM1 was 0.24 (95 %CI 0.02–2.74; *P* = 0.25) ( Table 2 ).

One recurrence was detected in the 38 HM0 cases versus 2/8 HM1 T1 CRC cases (2.6 % [95 %CI 0.14 %–13.5 %] vs. 25 % [95 %CI 7.2 %–59.1 %]), with an OR over time of 0.04 (95 %CI 0.004–0.42; *P* = 0.007) ( **Table 1 s** ).

**Fig. 2:**
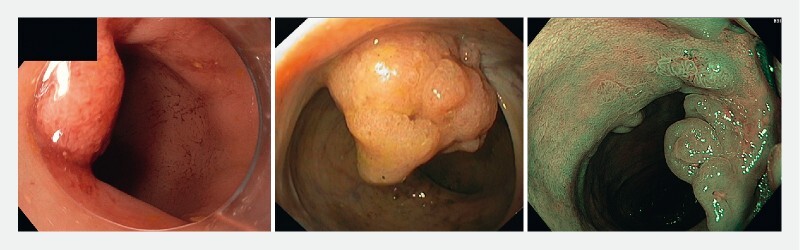
Example images of recurrent lesions on the scar after endoscopic submucosal dissection for a noninvasive lesion.

Details of all recurrences are displayed in Table 2 s, along with example images in Fig. 2 .

## Discussion

In this multicenter European cohort study, including the largest number of HM1 cases after complete colorectal ESD published so far, we found that HM1 was associated with a small absolute recurrence risk of 2.1 % in patients with ESD of a noninvasive polyp during a mean follow-up of 22 months, as compared with HM0 (0.5 %).

En bloc resections with EMR or cold snaring are associated with an overall recurrence rate of 3 % (95 %CI 2 %–5 %) 1 . This is close to zero in the HM0 resected group, but higher in HM1 resected neoplasms 10 . In contrast to these techniques, ESD permits visual control over the lateral resection margin during the incision, so is expected to result in a lower risk of local recurrence, even if the lateral resection margin is positive for dysplasia on histologic evaluation. Lee et al. showed that the 5-year cumulative recurrence rate following colorectal ESD in an HM1 resected group was 5 %, compared with 0.6 % in an HM0 resected group 6 ; however, the study included only 44 true HM1 resected cases. Furthermore, it was an Asian study, and these are known to show better outcomes for ESD than Western series. A higher recurrence rate of 4 % was shown in a recent meta-analysis on the long-term outcomes of ESDs performed in Europe 11 . However, this meta-analysis did not discriminate between HM0 and HM1 resected polyps, nor whether the ESD was en bloc or converted into a piecemeal resection.

In our study of 96 benign en bloc ESD HM1 resected cases, with similar median lesion size to Lee et al., we were able to confirm the low recurrence rate 6 . When the endoscopist is convinced of an en bloc resection, local recurrence risk is low despite pathologically positive horizontal margins. This finding challenges the recommendations made by the ESGE guideline for ESD and Park et al. to perform a colonoscopy at 3–6 months after HM1 and provides justification for postponing follow-up to at least 12 months 4 5 .

These findings question whether, within the R0 resection after an en bloc ESD, the horizontal resection margin should be addressed separately from the vertical resection margin. An outcome parameter should have clinical consequences, such as creating an indication for adjuvant treatment or differences in follow-up, whereas the finding of HM1 after a complete ESD does neither 6 . To increase the margin of evaluation for the pathologist, the distance to the outer margin of the lesion could be increased. This would however have significant consequences for the size of the resected specimen and therefore the duration and safety of the ESD, as well as the risk of stricture development. In noninvasive lesions, an HM0 resection should therefore be focused on en bloc resection and the deep resection margin. New strategies, such as the pocket-creation method or traction devices, could be used as they may result in higher en bloc and R0 resection rates of the vertical margin 12 13 14 .

This study has some important limitations. First, the median follow-up was 23.9 months for noninvasive lesions. Although the cumulative recurrence rates are known to be 96 % and 98 % at 6 and 12 months respectively, there is still a risk of late recurrence, up to 3 years, especially given recurrences in our study were detected at 17–46 months 1 . It is therefore not possible to exclude that the cumulative recurrence risk increases up to or above 5 % at 5 years, as was suggested in the study by Lee et al 6 . Both in their study and in that of Suchy et al., recurrences appear to be late 15 . That said, this would be an alternative argument for late follow-up, meaning at least no earlier than 12 months. In addition, a large group of patients in our study were lost to follow-up but, besides a shorter procedure duration, we found no differences at baseline between the included and excluded HM1 patients.

Second, the follow-up after the ESD was not standardized and was left to the discretion of the treating physician. As a result, not all cases had a follow-up endoscopy at predefined time points; however, because recurrence is not known to regress by itself, a negative finding at a later date can be extrapolated to a 6-month and 12-month period.

Third, 2/8 HM1 T1 CRC cases showed recurrence of cancer during follow-up, which was also significantly shorter than for HM0 cases. The number of HM1 T1 CRC cases was however small, resulting in a wide confidence interval of 7 %–59 %. Furthermore, the 25 % recurrence rate is significantly higher than the recurrence rate observed in patients with a positive margin for carcinoma 16 . It is questionable whether this reflects the actual risk of recurrence within T1 CRCs, and therefore this requires further study. Interestingly, both recurrences were T3 cancers despite negative vertical margins, possibly explained by other factors increasing the risk of recurrence, such as skip lymphovascular invasion or tumor budding beyond the resection margin 17 18 19 .

In conclusion, the risk of recurrence within 12 months after a complete ESD with positive horizontal resection margins is very low for noninvasive lesions. Postponing the first follow-up endoscopy to 12 months after the initial resection might be justified for noninvasive lesions.

## Tables 1 s, 2 s

Supplementary material Zusatzmaterial
