# Supplementary material for: Low risk of local recurrence after a successful en bloc endoscopic submucosal dissection for noninvasive colorectal lesions with positive horizontal resection margins (R-ESD study)
Source: Endoscopy. 2023 Jan 12;55(3):245–51. doi: 10.1055/a-1960-3552 (PMC9974332; doi:10.1055/a-1960-3552)
Supplement: Supplementary file 1 — Supplementary material [file 21466supmat_10-1055-a-1960-3552.pdf]

Supplementary material

Low risk of local recurrence after successful en bloc endoscopic submucosal dissection for noninvasive colorectal lesions with positive horizontal resection margins (R-ESD study)

Krijn J.C. Haasnoot, Francisco Baldaque-Silva, Arjun Koch, Mariana Figueiredo Ferreira, João Santos-Antunes, Emanuel Dias, Masami Omae, Laurelle van Tilburg, Hao Dang, Arnaud Lemmers, Jurjen J. Boonstra, Leon M.G. Moons

 | © 2022. The author(s).

Supplementary material

Table 1s: Recurrence during follow-up HM0 vs. HM1 for T1 CRC

Follow-up time and detected recurrence						
**T1 CRC**	**Median follow-up time in months [IQR]**	**p-value**	**Recurrence detected during follow-up**	**95% CI for proportion***	**Odds Ratio (95%CI)¥**	**p-value**
**Total**	21.0 [12.0-42.0]					
**HM0**	27.4 [13.5-45.3]	0.001	1/38 (2.6%)	0.14-13.5%	0.04 (0.004-0.42)	0.007
**HM1**	9.2 [7.2-13.1]		2/8 (25%)	7.15-59.1%	Reference	

Survival analysis			
**T1 CRC**	**6 months**	**12 months**	**18 months****
**HM0**			
Recurrence / patients at risk	0/35	0/31	0/25
Recurrence free survival (95%-CI)	100% (100-100%)	100% (100-100%)	100% (100-100%)
**HM1**			
Recurrence / patients at risk	0/7	2/5	2/3
Recurrence free survival (95%-CI)	100% (100-100%)	60% (29-100%)	60% (29-100%)

Legend: * 95% Confidence Interval for proportion calculated with Wilson score method
¥ Odds ratio calculated from Generalized Estimating Equation
**Max. follow-up time for T1 CRC patients with HM1 is 18 months
Abbreviations: HM1: horizontal resection margin positive or indeterminate for dysplasia; HM0: a free horizontal resection margin; IQR: Interquartile Range; CI: Confidence Interval; T1 CRC: T1 Colorectal Carcinoma

Supplementary material

Table 2s: Details of cases with recurrence during follow-up

Sex	Age	Location	Paris Classification	Size	Pathologic report of the original lesion	Horizontal margin status	Nr. of follow-up endoscopies till recurrence	Time to recurrence detection	Treatment of recurrence	Pathologic report
**Non-invasive lesions**										
Male	84	Rectum	IIa + Is	40mm	Villous adenoma with LGD	HM1	1	17 months	EMR	HGD
Male	49	Sigmoid	II-a	8mm	Tubular adenoma with LGD in IBD-patient	HM1	2	26 months	Colectomy for dysplasia in IBD	Tubulovillous adenoma
Female	71	Recto-sigmoid	Is	20mm	Tubulovillous adenoma LGD with focal HGD	HM0	2	27 months	EMR	LGD
**T1 CRC**										
Female	82	Rectum	IIa + Is	80mm	Tubulovillous adenoma with LGD and submucosal invasive carcinoma (sm3)*	HM1 for dysplasia (HM0 for invasive component)	2	9 months	Surgery	pT3N1
Female	82	Recto-sigmoid	IIa + Is	90mm	Tubular adenoma with LGD and submucosal invasive carcinoma (sm1)*	HM1 for dysplasia (HM0 for invasive component)	1	6 months	Surgery + Radiation	pT3N2
Male	63	Rectum	Is	80mm	Tubulovillous adenoma with HGD and submucosal invasive carcinoma (sm2)*	HM0 for dysplasia and invasive component	3	46 months	Patient wishes no treatment	cT3N+

* Low-risk T1 without lymphovascular invasion, low-grade tumor budding, and good/moderate differentiation grade.

Abbreviations: HM1: horizontal resection margin positive or indeterminate for dysplasia; HM0: a free horizontal resection margin; LGD: Low-grade Dysplasia; HGD: High-grade dysplasia; EMR: Endoscopic Mucosal Resection; IBD: Inflammatory Bowel Disease.
